# Clinical Lectures on Paralysis, Disease of the Brain, and Other Affections of the Nervous System

**Published:** 1855-11

**Authors:** 


ART. V.— *Clinical Lectures on Paralysis, Disease of the Brain, and other Affections of the Nervous System.* By ROBERT BENTLEY TODD, M. D., F. R. S., Physician to King's College Hospital. Philadelphia: Lindsay & Blakiston. 1855.

This is, we believe, the first appearance of Dr. Todd as author of a premeditated book; though he is well known to the reading public as the writer of many valuable fugitive papers on subjects connected with practical medicine.

The present volume is made up of clinical lectures. In remarks which we have hitherto made, depreciatory of clinical teaching as forming subject matter for books, we did not include that class of cliniques in which the case is merely brought in to illustrate enlarged views of the disease under comment—where it becomes the accessory and not the essential part of the lecture. In this form printed clinical lectures may be readable and valuable; in any other their value is confined to the class which listens.

We are happy to include Dr. Todd's book in the former category.

Under the head of paralysis, our author considers first that form produced by the poison of lead. In accounting for the great frequency of wrist-drop as the prevalent form, he ignores the usual explanation that the hands being most exposed to direct contact with the poison, their muscles and nerves are the first to suffer. Asking why it is that the muscular and nervous tissues are chiefly affected, why the extremities rather than the trunk, why the extensors rather than the flexors, he answers that the poison enters the circulation chiefly by the lungs, "that those tissues in which the nutrient changes are most active receive the largest proportional supply of blood, and that blood, being loaded by a poisonous material, would impregnate them with it to a greater degree than other tissues in which the circulation is less active; that for this reason such highly nourished structures as muscle and nerve become poisoned early; that as the muscles of the upper extremities

---

are used more, and probably on that account experience more active nutrient changes than those of the trunk and lower extremities, the former are poisoned first. Moreover, in painters, the extensor muscles, as well as the muscles constituting the ball of the thumb, become principally paralyzed, because they are most exercised during the practice of painting; and as they are more exercised are consequently more supplied with blood—poisoned blood—to repair the waste that is going on in them."

This explanation seems to us more satisfactory than the commonly received opinion.

In the treatment Dr. Todd states that he has seen much benefit result from the sulphur bath. His formula is from two to four ounces of the sulphuret of potassium, mixed with twenty or thirty gallons of water. The use of iodide of potassium, in large doses, as a means of elimination, is also recommended, and he also ascribes much virtue to galvanism.

Another case illustrates that form of hysterical paralysis which is so common. The diagnostic symptoms were the absence of cerebral trouble, the suddenness of the invasion, and especially the method in which the paralytic extremity was dragged along, instead of the circumduction incident to cerebral paralysis. In speaking of this form, Dr. Todd is remarkably clear in pointing out the distinctions between it and the more serious cerebral lesion.

All the cases described exhibit much shrewdness in diagnosis. Some of the differential symptoms of paralysis seem to us more forcibly presented than we have elsewhere seen. Tonic contraction of the muscles with decrease of size, or firmness with no diminution in bulk, are considered as indicative of inflammatory action and antiphlogistic treatment, while complete relaxation with little or no galvanic irritability present the opposite indications.

On apoplexy our author affirms that a vast number of the cases which occur about or subsequent to the fiftieth year of life depend upon atheromatous deposit in the arteries, a condition which holds out small inducement for the formerly universal practice of bloodletting. With reference to this question of depletion in apoplexy, he refers the student to Mr. Copeman's tables. "Of 155 cases 129 were bled, and only 26 were not. Of the 129 bled, 51 recovered, and 78 died—the recoveries being about 1 in $2\frac{1}{2}$, the deaths 1 in $1\frac{2}{3}$. Of the 26 not bled 18 survived, and 8 died, the proportion of recoveries being 1 in $1\frac{1}{2}$, and of deaths 1 in $3\frac{1}{4}$."

In no disease is the temptation to heroic measures more strong than in apoplexy, and in none is greater discrimination required as to the use of the lancet.

We have not space to follow Dr. Todd through his consideration of brain disease, renal coma, delirium, hemiplegia, (on which he is particularly full and satisfactory in the forms of epileptic, chronic, spinal and hysterical hemiplegia) of epileptic coma, lead palsy, syphilitic disease of the dura-mater, trismus, chorea, local hysteria and catalepsy. This catalogue embraces, we believe, all the subjects treated. We commend the book to our readers with a full confidence in its practical value at the bedside. It partially fills a vacuum in our works on practice, and is one of that now rapidly series of monographs on special subjects, which are so much better suited to the wants of the practitioner than the barren details to be found in works on general practice.

